# N6-Methylandenosine-Related lncRNAs Are Potential Biomarkers for Predicting the Overall Survival of Lower-Grade Glioma Patients

**DOI:** 10.3389/fcell.2020.00642

**Published:** 2020-07-23

**Authors:** Zewei Tu, Lei Wu, Peng Wang, Qing Hu, Chuming Tao, Kuangxun Li, Kai Huang, Xingen Zhu

**Affiliations:** ^1^Department of Neurosurgery, The Second Affiliated Hospital of Nanchang University, Nanchang, China; ^2^East China Institute of Digital Medical Engineering, Shangrao, China; ^3^Institute of Neuroscience, Nanchang University, Nanchang, China; ^4^College of Queen Mary, Nanchang University, Nanchang, China

**Keywords:** lower-grade glioma, N6-methylandenosine, long non-coding RNA, prognostic signature, ceRNA network

## Abstract

The prognostic value of N6-methylandenosine-related long non-coding RNAs (m6A-related lncRNAs) was investigated in 646 lower-grade glioma (LGG) samples from The Cancer Genome Atlas (TCGA) and the Chinese Glioma Genome Atlas (CGGA) datasets. We implemented Pearson correlation analysis to explore the m6A-related lncRNAs, and then univariate Cox regression analysis was performed to screen their prognostic roles in LGG patients. Twenty-four prognostic m6A-related lncRNAs were identified as prognostic lncRNAs and they were inputted in a least absolute shrinkage and selection operator (LASSO) Cox regression to establish a m6A-related lncRNA prognostic signature (m6A-LPS, including 9 m6A-related prognostic lncRNAs) in the TCGA dataset. Corresponding risk scores of patients were calculated and divided LGG patients into low- and high-risk subgroups by the median value of risk scores in each dataset. The m6A-LPS was validated in the CGGA dataset and it showed a robust prognostic ability in the stratification analysis. Principal component analysis showed that the low- and high-risk subgroups had distinct m6A status. Enrichment analysis indicated that malignancy-associated biological processes, pathways and hallmarks were more common in the high-risk subgroup. Moreover, we constructed a nomogram (based on m6A-LPS, age and World Health Organization grade) that had a strong ability to forecast the overall survival (OS) of the LGG patients in both datasets. We also establish a competing endogenous RNA (ceRNA) network based on seven of the twenty-four m6A-related lncRNAs. Besides, we also detected five m6A-related lncRNA expression levels in 22 clinical samples using quantitative real-time polymerase chain reaction assay.

## Introduction

Gliomas are obstinate intracranial tumors with very poor mortality and recurrence rates (Jiang et al., 2016). They are extremely difficult to remove by neurosurgical resection due to their invasiveness. Lower-grade gliomas (LGGs) can evolve to higher-grade gliomas and develop resistance to chemotherapy. These factors result in gliomas having a persistently high mortality rate. Therefore, searching for therapeutic targets for treating gliomas is urgent (The Cancer Genome Atlas (TCGA) Research Network, 2008; Brat et al., 2015; Jiang et al., 2016).

N6-methylandenosine (m6A) modification, the most abundant epigenetic methylated modification of messenger RNAs (mRNAs) and non-coding RNAs (ncRNAs), plays a vital role in RNA splicing, export, stability and translation (Zhao et al., 2017; Dai et al., 2018). m6A modification is a invertible and dynamical RNA epigenetic process that is regulated by m6A regulators, including “writers” (methyltransferases), “readers” (signal transducers) and “erasers” (demethylases) (Zaccara et al., 2019). Recent research had revealed that m6A modification can regulate oncogenesis and tumor progression in several kinds of cancers, including glioma. For example, METTL14 could enhances leukemia stem cells self-renewing and acts as a cancer promoter in the development and maintenance of acute myeloid leukemia (Weng et al., 2018); knockdown of FTO limits glioblastoma stem cells self-renewing and the proliferation and invasion of lung squamous cell carcinoma (SCC) cells (Cui et al., 2017; Liu et al., 2018); and YTHDF2 restrains cell proliferation by reducing the mRNA stability of EGFR in liver cancer (Zhong et al., 2019). Recently, bioinformatic research has revealed that dysregulation of m6A regulators involved in the malignant development of gliomas (Chai et al., 2019).

Aberrant lncRNA expression is also strongly related to tumor malignancy, and dysregulation of long non-coding RNAs had been confirmed to play a critical role in the pathogenicity of gliomas (Yang G. et al., 2014; Bhan et al., 2017). For instance, HOXA11-AS is reported to be a cell cycle-related lncRNA as well as a biomarker of glioma patients (Wang et al., 2016; Se et al., 2017); MALAT1 was a suppressor of glioma by downregulating MMP2 and devitalizing ERK/MAPK signaling (Han et al., 2016); and knockdown of DANCR could inhibit glioma cell growth and invasion through downregulating miR-135a-5p/BMI1 axis (Feng et al., 2020). However, the full role of m6A regulators in the dysregulation of lncRNAs in cancers remains unclear, and few studies have been performed to explore the mechanisms underlying how m6A modifications contribute to lncRNA-dependent glioma occurrence and development. Thus, understanding how m6A modifications of lncRNAs are involved in glioma progression may help to identify biomarkers that can act as useful therapeutic targets.

Here, based on The Cancer Genome Atlas (TCGA) dataset (*n* = 476) and the Chinese Glioma Genome Atlas (CGGA) dataset (*n* = 170), we identified the prognostic significance of m6A-related lncRNAs by bioinformatic and statistical analysis of data from patients with LGG. Our results showed that 24 m6A-related lncRNAs had prognostic value in both TCGA and CGGA LGG patients. Furthermore, we constructed an m6A-related lncRNA prognostic signature (m6A-LPS) based on the ability of nine m6A-related lncRNAs to predict the OS of LGG patients. In the meanwhile, LGG patients in low- and high-risk subgroups (categorized based on the m6A-LPS) had different prognosis and tumor hallmarks were more common in the high-risk subgroup. Furthermore, an accurate nomogram was constructed to predict OS in patients with LGG and a ceRNA network was built to search the target miRNAs and mRNAs of these m6A-related prognostic lncRNAs.

## Materials and Methods

### Datasets and m6A-Related Genes

For TCGA training set, mRNA expression files [Fragments Per Kilobase of transcript per Million mapped reads (FPKM) normalized] were acquired from the Genomic Data Commons Data Portal^1^ and the corresponding clinicopathological data was obtained from the cBioPortal website^2^. To obtain a CGGA validation set, RNA-seq data and related clinicopathological data were downloaded from the CGGA website^3^. Gliomas of World Health Organization (WHO) grade II and III were defined as lower-grade gliomas (LGGs), and LGG patients with missing OS values or OS < 30 days were excluded in order to reduce statistical bias in our analysis. Ultimately, we obtained a TCGA training dataset involving 476 patients and a CGGA validation dataset involving 170 patients. In addition, based on previous publications, expression matrixes of 21 m6A-related genes were extracted from the TCGA and CGGA databases, respectively, including expression data on writers (*METTL3, METTL14, METTL16, WTAP, VIRMA [KIA1499], RBM15, RBM15B, and ZC3H13*), erasers (*FTO* and *ALKBH5*) and readers (*YTHDC1, YTHDC2, IGF2BP1, IGF2BP2, IGF2BP3, YTHDF1, YTHDF2, YTHDF3, HNRNPC, HNRNPA2B1*, and *RBMX*). The clinicopathological and molecular features for the samples including in current study were collected in Supplementary Table S1.

### Annotation of lncRNAs

The long non-coding RNA annotation file of Genome Reference Consortium Human Build 38 (GRCh38) was acquired from the GENCODE website^4^ for annotation of the lncRNAs in the TCGA dataset. Based on recognizing the Ensemble IDs of the genes, 14,247 lncRNAs were identified in the TCGA dataset, and 4304 lncRNAs were identified based on the gene symbols in the CGGA dataset. In this part, lncRNAs defined in our analysis included eight types of transcripts (lincRNA, antisense, processed transcript, sense intronic, 3prime overlapping ncRNA, sense overlapping, and macro lncRNA).

### Bioinformatic Analysis

Pearson correlation analysis was first implemented to mining m6A-related lncRNAs (with the | Pearson R| > 0.5 and *p* < 0.001) in each dataset for finding m6A-related lncRNAs. Then univariate Cox regression analysis was implemented to filtrate the prognostic m6A-related lncRNAs in the two datasets, respectively. The m6A-related prognostic lncRNAs screened from the two databases are intersected to obtain the 24 shared m6A-related prognostic lncRNAs. Thereafter, using the R package “glmnet” (Friedman et al., 2010) to conduct least absolute shrinkage and selection operator (LASSO) Cox regression (with the penalty parameter estimated by 10-fold cross-validation) (Tao et al., 2020), we developed a m6A-related lncRNA prognostic signature (m6A-LPS) for the LGG patients involving 9 m6A-related lncRNAs. The risk score calculating formula is:

$$R\,i\,s\,k\,s\,c\,o\,r\,e=\sum_{i=1}^{n}C\,o\,e\,f_{i}*x_{i}$$

where *Coef*_*i*_ means the coefficients, *x*_*i*_ is the FPKM value of each m6A-related lncRNAs.

Risk scores were computed for all patients including in our study. For both two datasets, principal component analysis (PCA) was performed using R programming language (verison3.6.1) (see footnote 5) and scatter diagrams were plotted using the R package “ggplot2.” Using the TCGA cohort, differentially expressed genes (DEGs) in the high-risk subgroup in contrast to the low-risk subgroup were identified based on the standards of | log2(Fold change)| > 1 and *p* < 0.05 using the R package “limma” (Ritchie et al., 2015). Perl programming language was used to perform the prediction analysis of the target miRNAs of the 7 m6A-related lncRNAs in the miRcode database^5^ and 59 shared target mRNAs of these miRNAs was found in the miRTarBase^6^, miRDB^7^, and TargetScan^8^ databases. Respectively, the 2571 differential expression genes (DEGs) between low- and high-subgroups and the 59 target mRNAs in the ceRNA network were then inputted into the “Metascape” website (Zhou et al., 2019)^9^ for functional and pathway enrichment analysis, which involved Canonical Pathways, Reactome Gene Sets, Gene Ontology (GO) Biological Processes and Kyoto Encyclopedia of Genes and Genomes Pathway (KEGG pathway). Additionally, we used GSEA software^10^ to investigate the tumor hallmarks that were more common in the high-risk subgroup compared with the low-risk subgroup. The ceRNA network was plotted using the software of “Cytoscape” (Shannon et al., 2003).

### Samples and Quantitative Real-Time Polymerase Chain Reaction (qRT-PCR)

We totally collected 22 non-neoplastic and neoplastic samples from patients who underwent surgical treatments in the Neurosurgical Department of The Second Affiliated Hospital of Nanchang University from 2015 to 2019. In these 22 clinical samples, 16 glioma samples were classified into 8 WHO grade II gliomas and 8 WHO grade III gliomas, and 6 non-neoplastic brain tissues (NBTs) were obtained from intractable epilepsy patients. Fresh tumor and non-neoplastic brain tissues were frozen and stored at -80°C. This research was approved by the Medical Ethics Committee of The Second Affiliated Hospital of Nanchang University and the sample acquisition and usage was performed in accordance with the approved guidelines. Informed consent was acquired from each involved patient.

For evaluating the expression levels of m6A-related lncRNAs, we extracted total RNA from clinical glioma samples by using RNA trizol reagent (Invitrogen, Carlsbad, CA, United States). According to the instructions of manufacturer, cDNA synthesis was carrying out by using reverse transcription kit (Guangzhou Ribobio Co., Ltd.). The qRT-PCR analysis was conducted on The LightCycler 480 Real-Time PCR System. Related lncRNA expression levels were calculated using the 2-ΔΔCT method and the related GAPDH mRNA expression was used as an endogenous control. Primers sequences used in our study were as follows: LINC00237 forward 5′-GCAGGCCCAGACTGTC AT-3′, and reverse 5′-ACTGGCCGGAGACCATTTG-3′; LINC 00925 forward 5′- AGGGACTCCAGCCTCTTAGG -3′, and reverse 5′-TGGGCCTTTTCCCTGCATAG-3′; LINC00265 forward 5′-GCAGGCCCAGACTGTCAT-3′, and reverse 5′-AC TGGCCGGAGACCATTTG-3′; GDNF-AS1 forward 5′-AACA GGCAAACACAAGGTGC-3′, and reverse 5′-GCTTGCAGTGT GATGTTGGG-3′; C6orf3 forward 5′-TCCTTCACGCCAT CACAAGA-3′, and reverse 5′-TTTGTTGGTGCTCTGTC AACC-3′.

### Statistical Analyses

Kaplan–Meier curves and the log-rank test were used to compare the OS between various subgroups, comprising the low- and high- risk subgroups and additional subgroups based on the expression of each of the nine m6A-related lncRNAs. The student’s *t*-test was used to compare the risk scores (based on the m6A-LPS) between pairs of subgroups based on the following clinicopathological features: isocitrate dehydrogenase (IDH) mutation status (mutant or wildtype IDH), 1p/19q co-deletion status (co-deletion or non-co-deletion), O(6)-methylguanine-DNA methyltransferase (MGMT) promoter methylation status (methylated or unmethylated), age (≤ 40 or > 40 years), WHO grade (II or III) and gender (male or female). Chi-square tests were used to compare the distribution of age, gender, WHO grade, IDH status, and 1p/19q status between low- and high-risk subgroups (partitioned by the median value of risk scores) in LGGs of both datasets (Supplementary Table S2). Univariate and multivariate Cox regression analyses were utilized to evaluate the independent prognostic value of the m6A-LPS regarding OS.

We performed multivariate Cox regression to establish a nomogram, the calibration plots showedthe prognostic predictive accuracy of the nomogram and the C-index was calculated for the nomogram models in both two datasets. These analyses were performed using the R package “rms.” The prognostic ability of the nomogram and other predictors (risk score, age, and WHO grade) for 1/3/5-year OS was evaluated by receiver operating characteristic (ROC) curves (R package “timeROC”) and the area under the curve (AUC) values (Blanche et al., 2013). The statistical analysiscarried out in this study was using R programming language (version 3.6.1)^11^ and SPSS Statistics 25 software^12^. The related R codes were uploaded to the git-hub repository^13^.

## Results

### Identification of m6A-Related lncRNAs in LGG Patients

**FIGURE 1:**
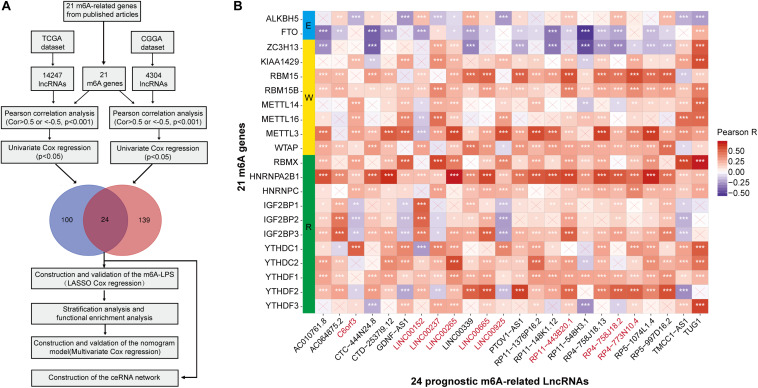
**(A)** Study flow chart. **(B)** Heatmap of the correlations between m6A-related genes and the 24 prognostic m6A-related lncRNAs. *p < 0.05, **p < 0.01, and ***p < 0.001.

Firstly, using the downloaded file from the “GENCODE” website, we identified 14247 lncRNAs in the TCGA dataset and 4304 lncRNAs in the CGGA dataset for the following analysis. We then extracted the expression matrixes of 21 m6A-related genes from the TCGA and the CGGA datasets, respectively. A lncRNA whose expression value was correlated with one or more of the 21 m6A-related genes (| Pearson R| > 0.5 and *p* < 0.001) was defined as a m6A-related lncRNA. Pearson correlation analysis was performed to search m6A-related lncRNAs in each dataset, and we obtained 75 lncRNAs which were significantly correlated with m6A-related genes in both two datasets. Combined with the prognostic information, univariate Cox regression was then implemented to screen m6A-related prognostic lncRNAs from the 75 m6A-related lncRNAs in both the TCGA and CGGA datasets (*p* < 0.05), respectively. Finally, we found that 24 m6A-related lncRNAs were significantly correlated with the OS of LGG patients in both two datasets. The work flow was shown in Figure 1A and the correlations between the 24 lncRNAs and the m6A-related genes in the TCGA dataset are shown in Figure 1B. The results of univariate Cox analysis of the twenty-four m6A-related lncRNAs are shown in Table 1.

**TABLE 1:** The twenty-four m6A-related prognostic lncRNAs.

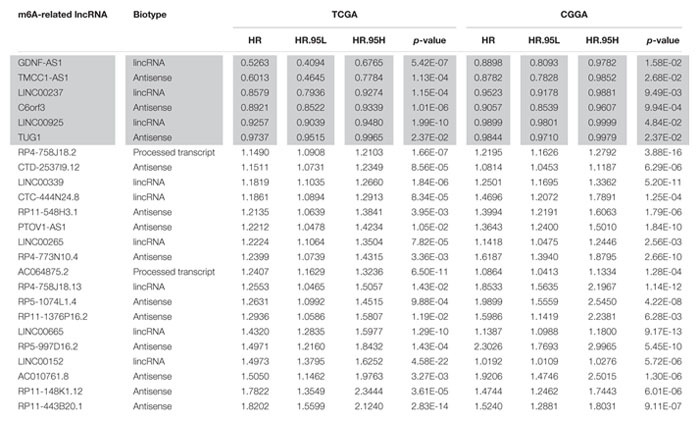

*Color shaded lncRNAs were protective lncRNAs and others were risky lncRNAs.*

### Construction of the m6a-LPS in the TCGA Dataset

**FIGURE 2:**
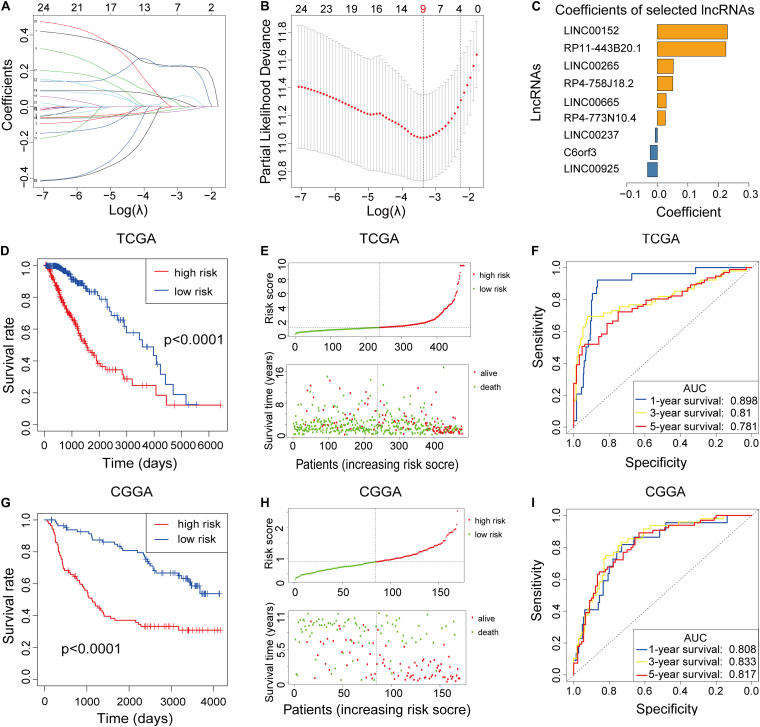
**(A–C)** Least absolute1 shrinkage and selection operator (LASSO) regression was performed, calculating the minimum criteria **(A,B)** and coefficients **(C)**. **(D)** Kaplan–Meier curves showed that the high-risk subgroup had worse overall survival than the low-risk subgroup in The Cancer Genome Atlas (TCGA) dataset. **(E)** Distributions of risk scores [based on the m6A-related lncRNA prognostic signature (m6A-LPS)] and survival status of LGG patients in the TCGA dataset. **(F)** Receiver operating characteristic (ROC) curves of m6A-LPS for predicting the 1/3/5-year survival in the TCGA dataset. **(G)** Kaplan–Meier curves showing that the high-risk subgroup had worse overall survival than the low-risk subgroup in the Chinese Glioma Genome Atlas (CGGA) dataset. **(H)** Distributions of risk scores and survival status of LGG patients in the CGGA dataset. **(I)** ROC curves of m6A-LPS for predicting 1/3/5-year survival in the CGGA dataset.

To build the m6A-LPS for forecasting the OS of LGG patients, we performed a LASSO Cox analysis on the basis of the 24 m6A-related prognostic lncRNAs in the TCGA cohort and it generated the m6A-LPS which contains 9 m6A-related lncRNAs and coefficient of each (Figures 2A,B). The m6A-LPS involved nine lncRNAs and, for each patient in the TCGA dataset, a risk score was calculated based on the coefficient for each lncRNA (Figure 2C). Patients in the TCGA cohort were divided into low- and high-risk subgroups based on the median value of risk scores. Kaplan–Meier survival curves depicted that LGG patients with higher risk scores had worse clinical outcomes (lower OS rates and a shorter OS time) (Figure 2D). Risk score and survival status distributions are plotted in Figure 2E. And the ROC curves demonstrated that m6A-LPS harbored a promising ability to predict OS in the TCGA cohort (1-year AUC = 0.898, 3-year AUC = 0.810, 5-year OS = 0.781; Figure 2F).

### Validation of the m6a-LPS in the CGGA Dataset

To validate the prognostic ability of m6A-LPS, we calculated risk scores for patients in the CGGA cohort using the same formula. LGG patients in the CGGA dataset were assigned to low- and high-risk groups based on the median risk score. The results were consistent with the findings in the TCGA dataset: LGG patients with higher risk scores had lower OS rates and a shorter OS time in the CGGA dataset (Figure 2G). Risk score and survival status distributions are shown in Figure 2H and it showed that patients with higher risk scores had shorter overall survival time and dead status. The ROC analysis also indicated that m6A-LPS had a strong prognostic value for LGG patients in the CGGA dataset (1-year AUC = 0.808, 3-year AUC = 0.833, 5-year AUC = 0.817; Figure 2I). These results showed that the m6A-LPS had a robust and stable OS-predictive ability.

### Prognostic Analysis of the Nine m6A-Related lncRNAs

**FIGURE 3:**
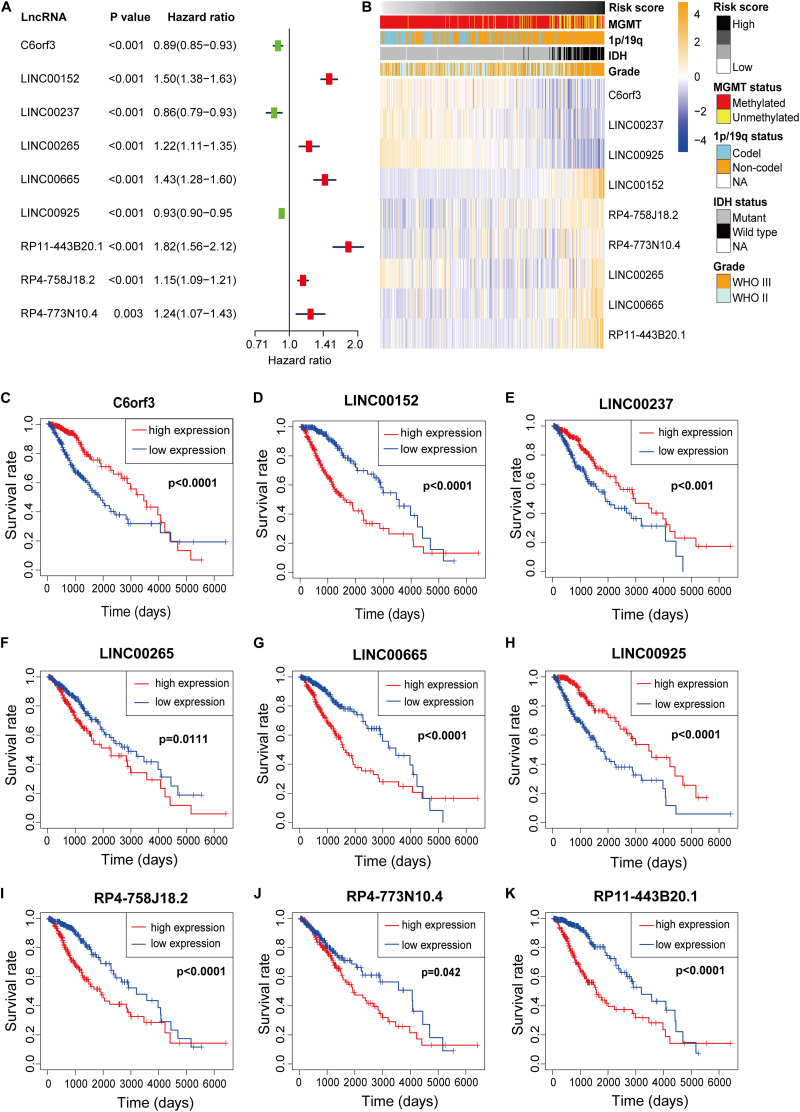
**(A)** Forest plot of the prognostic ability of the nine m6A-related lncRNAs included in the prognostic signature. **(B)** Heatmap of the associations between the expression levels of the nine m6A-related lncRNAs and clinicopathological features in the TCGA dataset. **(C–K)** Kaplan–Meier curves showing that patients with different expression levels of the nine m6A-related lncRNAs had different overall survival.

Nine m6A-related lncRNAs were included in the m6A-LPSand univariate Cox regression analysis was used to evaluate their prognostic roles. The forest plot shows that C6orf3, LINC00237 and LINC00925 are protective factors with HR (Hazard ratio) < 1, while LINC00152, LINC00265, LINC00665, RP11-443B20.1, RP4-758J18.2, and RP4-773N10.4 are risk factors with HR < 1 in LGG patients (Figure 3A). The heatmap (Figure 3B) shows that C6orf3, LINC00237, and LINC00925 expression decreased with increasing risk score, whereas the expression of the LIN00152, LINC00265, LINC00665, RP11-443B20.1, RP4-758J18.2, and RP4-773N10.4 increased with increasing risk score. Their expression levels were also related to the clinicopathological features of glioma, such as IDH mutation status, 1p/19q co-deletion status, MGMT methylation status and WHO grade (Figure 3B). The Kaplan–Meier survival curves confirmed that higher expression of C6orf3, LINC00237 and LINC00925 and lower expression of LIN00152, LINC00265, LINC00665, RP11-443B20.1, RP4-758J18.2, and RP4-773N10.4 were associated with better OS in the TCGA dataset (Figures 3C–K).

### Stratification Analysis of the m6a-LPS

**FIGURE 4:**
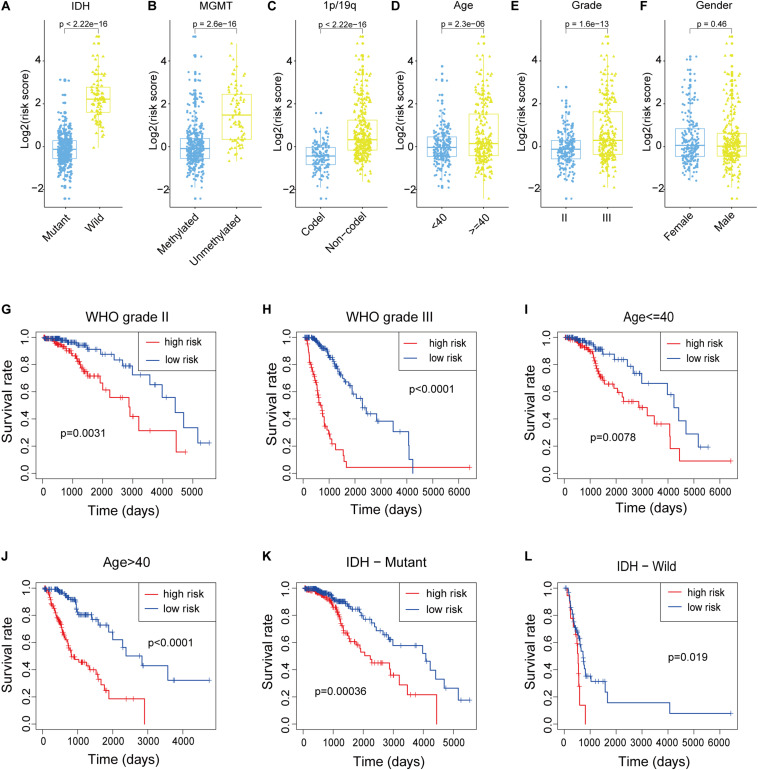
**(A–F)** Patients with different clinicopathological features (including IDH mutation status, 1p/19q co-deletion status, MGMT methylation status, age and WHO grade, but not gender) had different levels of risk scores, calculated based on the m6A-related lncRNA prognostic signature (m6A-LPS). **(G–L)** The m6A-LPS retained its prognostic value in multiple subgroups of LGG patients (including patients with WHO grade II or III, patients aged ≤ 40 or > 40 years, and patients with mutant or wildtype IDH). *p < 0.05, **p < 0.01, ***p < 0.001, and ****p < 0.0001.

We attempted to identify whether clinicopathological features were associated with the risk score. The results revealed that LGG patients with wildtype IDH, 1p/19q non-co-deletion status, unmethylated MGMT, older age and WHO grade III (Figures 4A–E) had higher risk scores, while the risk score was not associated with gender (Figure 4F). To better assess the prognostic ability of the m6A-LPS, we performed a stratification analysis to confirm whether it retains its ability to predict OS in various subgroups. In contrast with patients with lower risk, higher risk LGG patients had worse OS in both the WHO grade II and III subgroups (Figures 4G,H). Likewise, we confirmed that m6A-LPS retained its ability to predict OS for patients aged ≤ 40 or > 40 years (Figures 4I,J) and patients with mutant or wildtype IDH (Figures 4K,L). These data indicated that it could be a potential predictor for LGG patients. Besides, expression levels of the nine m6A-related lncRNAs between primary and recurrent LGGs were compared in the CGGA dataset (all patients in the TCGA cohort were primary LGGs). The result showed that C6orf3 was downregulated while LINC00152 was upregulated in current LGGs and other m6A-related lncRNAs were not differentially expressed between primary and current LGGs (Supplementary Figure S1A).

### Principal Component Analysis

The nine prognostic lncRNAs and their correlated m6A-related genes (m6A regulators) are shown in an alluvial diagram (Supplementary Figure S1B). Most of the correlated m6A regulators were m6A “readers”, and no “erasers” were statistically significant associated with the nine prognostic lncRNAs. Based on the expression value of the 21 m6A-related genes, principal component analysis (PCA) was performed to assess the differences between the low- and high-risk subgroups (Supplementary Figures S1C,D). The results showed that the low- and high-risk patients in both the TCGA and CGGA datasets were distributed in distinct directions. These results may suggest that differential m6A statuses exist in different risk subgroups.

### Pathway and Process Enrichment Analysis and Gene Set Enrichment Analysis (GSEA)

**FIGURE 5:**
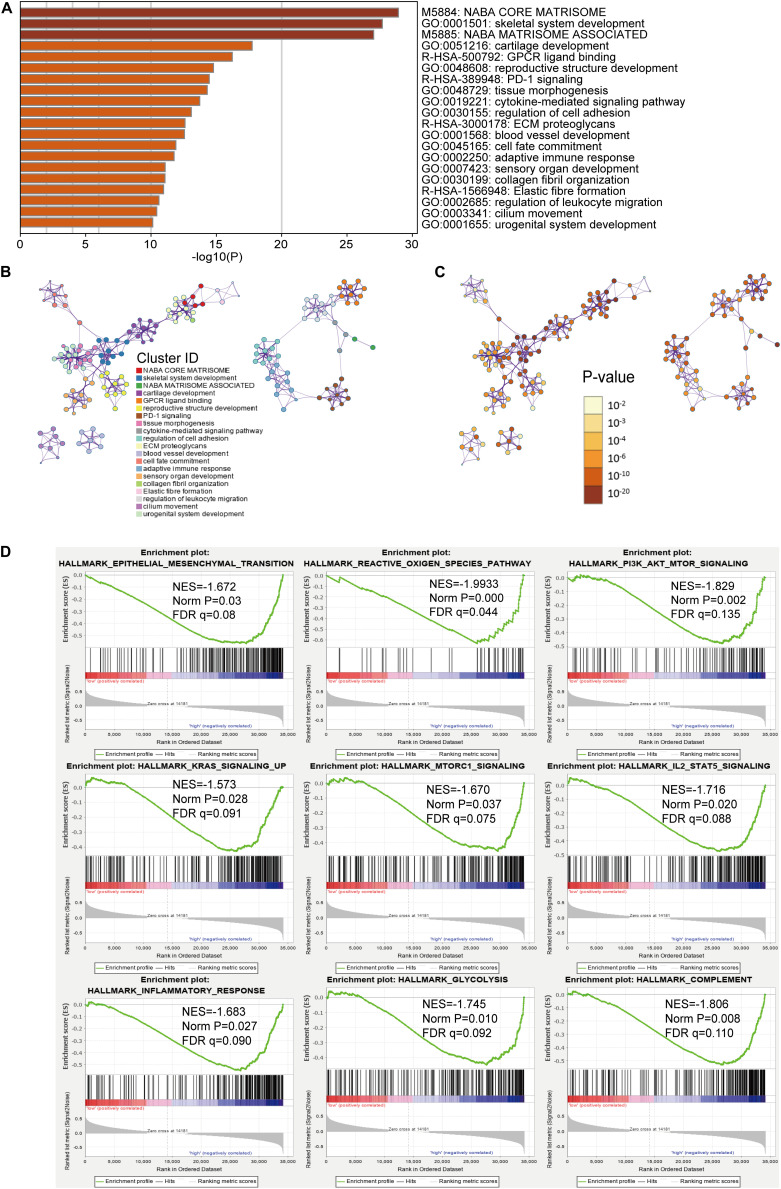
Functional analysis of 2571 differentially expressed genes (DEGs) between the low- and high-risk subgroups. **(A)** Heatmap of enriched terms across the inputted gene list, colored according to *p*-value. Network of enriched terms colored according to **(B)** cluster ID (nodes with the same cluster ID are typically close to each other) and **(C)** *p*-value (terms with more genes tend to have higher *p*-values). **(D)** Gene set enrichment analysis (GSEA) indicating that tumor hallmarks were enriched in the high-risk subgroup.

For investigating the potential biological process and pathway involving in the molecular heterogeneity between the low- and high-risk subgroups, we identified 2571 differential expression genes (DEGs) [|log2 (fold change)| > 1 and *p* < 0.05] between the low- and high-risk subgroups in the TCGA cohort. These DEGs were primarily enriched in these terms: NABA core matrisome, NABA matrisome-associated (Canonical Pathways); GPCR ligand binding, PD-1 signaling, ECM proteoglycans, elastic fiber formation (Reactome Gene Sets); cytokine-mediated signaling pathway, regulation of cell adhesion, blood vessel development and adaptive immune response (GO Biological Processes) (Figures 5A–C). Gene set enrichment analysis revealed that several tumor hallmarks were enriched in the high-risk subgroup, such as epithelial–mesenchymal transition, the reactive oxygen species pathway, P13K-AKT-MTOR signaling, inflammatory response, KRAS signaling, complement, IL2-STAT5 signaling, glycolysis and MTORC1 signaling (Figure 5D) and so on. These results may give us some insights into the cellular biological effects related to the m6A-LPS.

### m6A-LPS Was an Independent Prognostic Factor for LGG Patients

**FIGURE 6:**
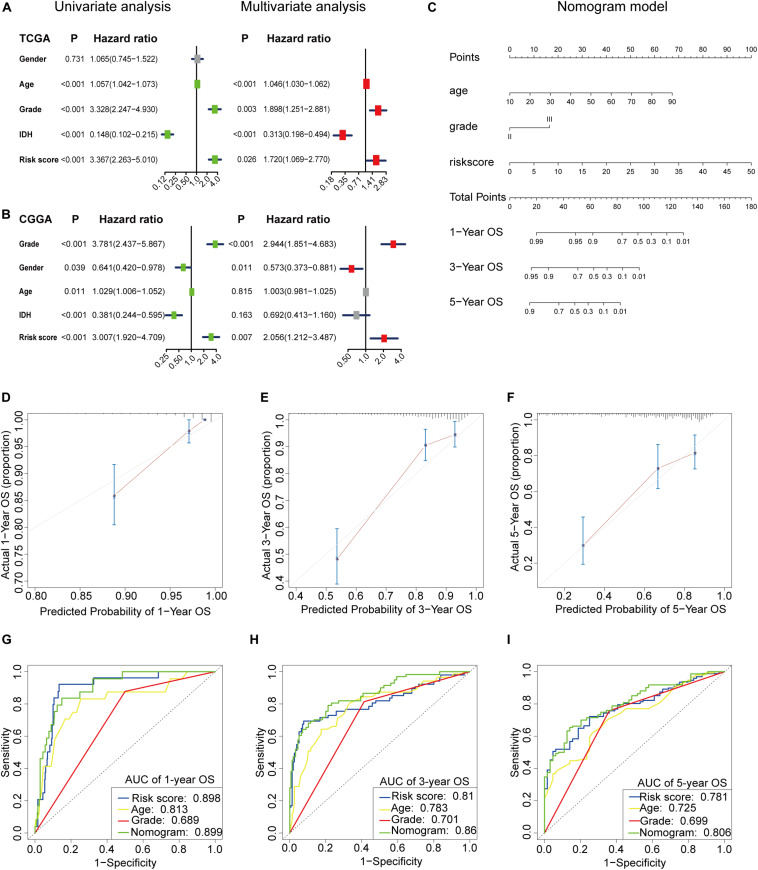
**(A,B)** Univariate and multivariate analyses revealed that risk score [based on the m6A-related lncRNA prognostic signature (m6A-LPS)] was an independent prognostic predictor in the TCGA and CGGA datasets. **(C)** Nomogram based on risk score, age and WHO grade. **(D–F)** Calibration plots of the nomogram for predicting the probability of OS at 1, 3, and 5 years in the TCGA dataset. **(G–I)** Time-dependent receiver operating characteristic (ROC) curves for the nomogram, risk score, age and grade in the TCGA dataset (for predicting 1, 3, and 5-year OS).

We used univariate and multivariate Cox analyses to assess whether the m6A-LPS was an independent prognostic factor for patients with LGG. Based on the data of LGG patients in the TCGA dataset, univariate Cox analysis indicated that m6A-LPS was remarkably associated with OS [hazard ratio (HR): 3.367, 95% CI: 2.263–5.010, *p* < 0.001; Figure 6A] and multivariate Cox analysis further showed that m6A-LPS was an independent predictor of OS (HR: 1.720, 95% CI: 1.069–2.770, *p* = 0.026; Figure 6A). The conclusion was validated in the CGGA dataset, which confirmed that m6A-LPS was an independent predictor of OS for LGG patients in the CGGA validation dataset (univariate: HR: 3.007, 95% CI: 1.920–4.709, *p* < 0.001; multivariate: HR: 2.056, 95% CI: 1.212–3.487, *p* < 0.001; Figure 6B). These results indicated that our m6A-LPS, as an independent prognostic indicator, might be useful for clinical prognosis evaluation.

### Construction and Validation of the m6A-LPS-Based Nomogram

To create a clinically applicable quantitative tool to predict the OS of LGG patients, we established a nomogram using the risk status (based on m6A-LPS), WHO grade and age in the TCGA dataset and it was also tested in the CGGA dataset (Figure 6C). Calibration plots showed that the observed vs. predicted rates of 1-, 3- and 5-year OS showed perfect concordance in the TCGA (Figures 6D–F) and CGGA cohorts (Supplementary Figure S2A). Then time-dependent ROC curves were used to assess the prognostic predictive ability of the nomogram and other predictors (risk score, age and WHO grade) in the TCGA (Figures 6G–I) and CGGA datasets (Supplementary Figure S2B and the results revealed that, compared with the other predictors, the nomogram had excellent accuracy regarding 1-, 3- and 5-year OS (AUC = 0.899, 0.860, and 0.806, respectively). C-index was also calculated for assessing the predictive ability of the nomogram in both two datasets as and results showed a stable and robust predictive power (C-index for the TCGA dataset: 0.817 and CGGA dataset: 0.642). These data indicated that the nomogram has a robust and stable ability to predictive the OS for LGG patients.

### Construction of the ceRNA Network and Functional Enrichment Analysis

**FIGURE 7:**
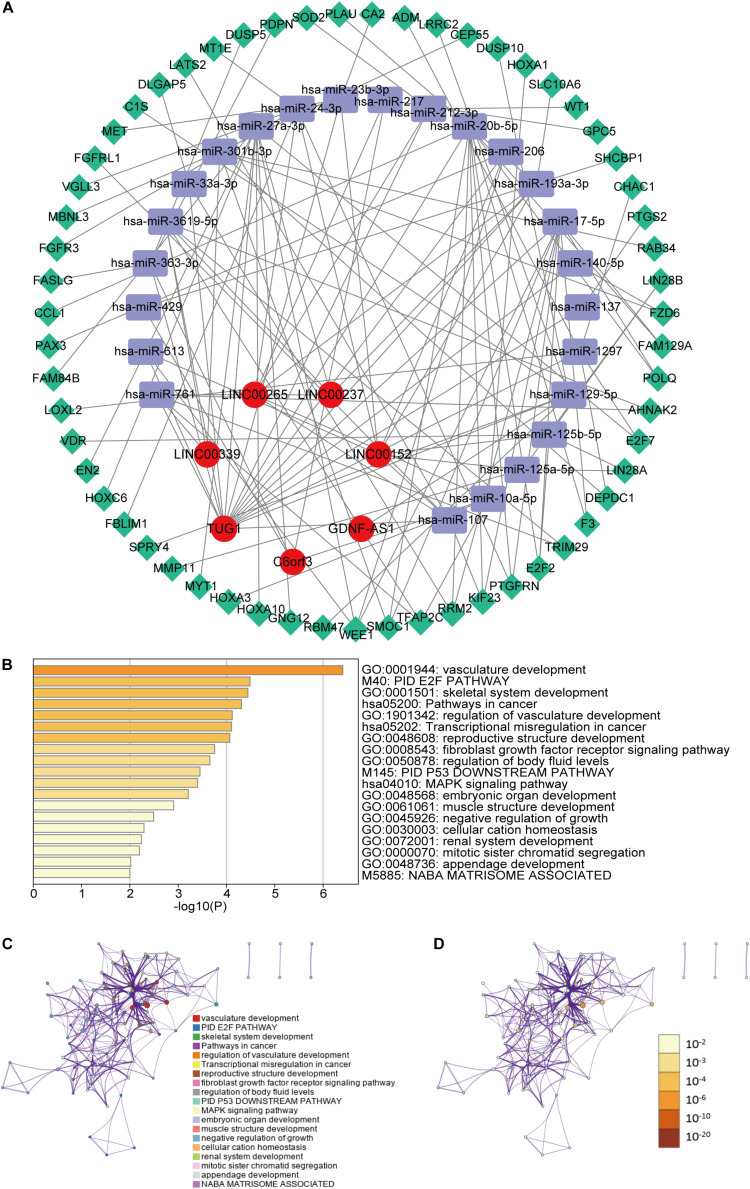
**(A)** The ceRNA network of the seven m6A-related lncRNAs (red) and their target miRNAs (blue) and mRNAs (green). **(B)** Heatmap of enriched terms across the fifty-nine mRNAs, colored according to *p*-value. Network of enriched terms colored according to **(C)** cluster ID (nodes with the same cluster ID are typically close to each other) and **(D)** *p*-value (terms with more genes tend to have higher *p*-values).

To further understand how the m6A-related lncRNAs regulate mRNA expression by sponging miRNAs in LGG, we constructed a ceRNA network based on the m6A-related lncRNAs. Seven of twenty-four lncRNAs were extracted from the miRcode database and 351 pairs of interaction between the seven lncRNAs and twenty-four miRNAs were identified. Then we used three databases (miRTarBase, miRDB, and TargetScan) to search target mRNAs based on the twenty-four miRNAs and totally fifty-nine mRNAs were identified in all the three databases. Ultimately, seven lncRNAs, twenty-four miRNAs and fifty-nine mRNAs were included in our ceRNA network (Figure 7A). Furthermore, the 59 target mRNAs were used to implemented functional enrichment analysis in the Metascape online tool and we found that these genes were enriched in vasculature development, fibroblast growth factor receptor signaling pathway, negative regulation of growth, mitotic sister chromatid segregation (GO Biological Processes); PID E2F pathway, PID P53 downstream pathway, NABA matrisome associated (Canonical Pathways); pathways in cancer, transcriptional misregulation in cancer, MAPK signaling pathway(KEGG Pathway) (Figures 7B–D). These data may provide us some clues for finding the potential functions of these m6A-related lncRNAs in LGGs.

### Validation of the Expression Levels of Five of the m6A-Related lncRNA in Glioma Samples

For validating the expression levels of the m6A-related prognostic lncRNAs in glioma samples, we detected five m6A-related prognostic lncRNAs expression levels in our collected 22 non-neoplastic and neoplastic samples including 16 glioma samples (8 WHO grade II gliomas and 8 WHO grade III gliomas) and 6 non-neoplastic brain tissues (NBTs) by using RT-qPCR assay. Our results showed that C6orf3, GDNF-AS1, LINC00237, and LINC00925 was downregulated in LGG samples compared with normal brain tissues and LINC00265 was upregulated in WHO grade III gliomas (Supplementary Figure S2C).

## Discussion

A total of 646 LGG patients from the TCGA and CGGA datasets were included in our study to exploit the prognostic significance of m6A-related lncRNAs. Twenty-four m6A-related lncRNAs were confirmed to have prognostic value in both the TCGA and CGGA datasets, and nine of them were used to establish an m6A-LPS for predicting the OS of LGG patients. Based on the median risk score, LGG patients were divided into the low- and high-risk subgroups, and the high-risk group had worse clinical outcomes and enrichment of tumor hallmarks and certain malignant related pathways. Multivariate Cox regression analysis showed that m6A-LPS was an independent risk factor for OS. Furthermore, combining m6A-LPS with age and WHO grade, we created a nomogram, and it had a robust ability to predict OS in LGG patients in the TCGA and CGGA datasets. A ceRNA network consist of seven m6A-related lncRNAs, twenty-four miRNAs and fifty-nine mRNAs were established for viewing the potential functions of these m6A-related lncRNAs. Finally, RT-qPCR was used to detected five m6A-related prognostic lncRNA expression levels in total 22 gliomas and normal brain tissues.

Multiple studies have suggested that m6A modification might function as a regulator in cancer pathogenesis, but how it acts in a lncRNA-dependent manner during glioma progression is still unknown. m6A regulators can maintain the malignancy of several kinds of tumors by modifying specific lncRNAs. Negatively controlled by YTHDF3, the lncRNA GAS5 could restrain colorectal cancer progression by causing YAP (Yes1 associated transcriptional regulator) phosphorylation and degradation (Ni et al., 2019). KIAA1429 [also named as Vir like M6A methyltransferase associated (VIRMA)] promotes liver cancer progression via m6A modification of the lncRNA GATA3 (Lan et al., 2019). METTL14 limits malignant progression of colon cancer by inhibiting the lncRNA XIST (Yang X. et al., 2020). Furthermore, lncRNA FOXM1-AS could accelerate the mutual effect of ALKBH5 and FOXM1 nascent transcripts and conduce to the maintenance of glioblastoma stem cells (Zhang et al., 2017). Study had revealed that m6A modification of lncRNAs could influence cancer initiation and progression, and lncRNAs might serve as competing endogenous RNAs, targeting m6A regulators and thereby influencing tumor aggressive progression. Taking the evidence together, we believe that m6A modification is targeted at lncRNAs, and we ought to pay more attention to the interactions and functions of lncRNAs and m6A modifications so as to identify potential prognostic markers or therapeutic targets of cancers.

We identified 24 m6A-related prognostic lncRNAs from 646 LGG patients, and nine of them were included in the m6A-LPS. LINC00152 is highly expressed and acts as an oncogene in colorectal cancer (Ou et al., 2020), and it may enhance malignancy and may be a promising prognostic biomarker of cancers (Seo et al., 2019; Wang et al., 2020). LINC00265 helps ZMIZ2 to stabilize β-catenin by acting as a sponge (and thereby inhibiting several miRNAs) in colorectal cancer (Zhu et al., 2019), and it can be used to predict poor prognosis in acute myeloid leukemia patients (Ma et al., 2018). It has been reported that LINC00665 increases the malignancy of hepatocellular carcinoma by activating the protein kinase R (PKR)/nuclear factor (NF)-κB pathway (Ding et al., 2020) and induce gastric cancer progression by activating the Wnt signaling pathway (Yang G. et al., 2014). Several of the nine lncRNAs were reported to be associated with cancer progression, but there have been few reports regarding glioma, and reports on how the lncRNAs interact with m6A-related genes have been even rarer. Thus, we hope that our results help to identify the prognostic lncRNAs that m6A regulators might target, thereby providing insights into their potential roles in LGG tumorigenesis and progression.

This study included two glioma datasets, the TCGA and CGGA datasets, and our results were derived and validated using them, but there were several limitations in our study. More independent glioma cohorts should be used to validate the identified prognostic m6A-related lncRNAs. Additionally, the roles of the lncRNAs and their interactions with m6A-related genes should be confirmed using *in vitro* and *in vivo* experiments. Our results may provide some clues for further researchs, concentrating on the mechanism process underlying m6A modification of lncRNAs.

## Data Availability Statement

Publicly available datasets were analyzed in this study. This data can be found here: http://cancergenome.nih.gov/, http://www.cgga.org.cn/.

## Author Contributions

XZ constructed this study. ZT, KH, and LW performed the data analysis, figures plotted, and writing. PW did the polymerase chain reaction experiments. CT, QH, and KL were responsible for the data acquisition and critical reading of the manuscript. All authors read and approved the final manuscript.

## Conflict of Interest

The authors declare that the research was conducted in the absence of any commercial or financial relationships that could be construed as a potential conflict of interest.

## Footnotes

1 https://portal.gdc.cancer.gov/

2 https://www.cbioportal.org/

3 http://www.cgga.org.cn/

4 https://www.gencodegenes.org/human/

5 http://www.mircode.org/

6 http://mirtarbase.mbc.nctu.edu.tw/php/index.php

7 http://mirdb.org/

8 http://www.targetscan.org/

9 http://metascape.org/

10 http://software.broadinstitute.org/gsea/index.jsp

11 https://www.r-project.org/

12 https://www.ibm.com/products/software

13 https://github.com/tzw2019/m6A-LPS-R

## References

[B1] Bhan A. Soleimani M. Mandal S. S. (2017). Long noncoding RNA and cancer: a new paradigm. *Cancer Res.* 77 3965–3981. 10.1158/0008-5472.can-16-2634 28701486 PMC8330958

[B2] Blanche P. Dartigues J. F. Jacqmin-Gadda H. (2013). Estimating and comparing time-dependent areas under receiver operating characteristic curves for censored event times with competing risks. *Stat. Med.* 32 5381–5397. 10.1002/sim.5958 24027076

[B3] Brat D. J. Verhaak R. G. Aldape K. D. Yung W. K. Salama S. R. Cooper L. A. (2015). Comprehensive, integrative genomic analysis of diffuse lower-grade gliomas. *N. Engl. J. Med.* 372 2481–2498. 10.1056/nejmoa1402121 26061751 PMC4530011

[B4] Chai R. C. Wu F. Wang Q. X. Zhang S. Zhang K. N. Liu Y. Q. (2019). m(6)A RNA methylation regulators contribute to malignant progression and have clinical prognostic impact in gliomas. *Aging* 11 1204–1225. 10.18632/aging.101829 30810537 PMC6402513

[B5] Cui Q. Shi H. Ye P. Li L. Qu Q. Sun G. (2017). m(6)A RNA methylation regulates the self-renewal and tumorigenesis of glioblastoma stem cells. *Cell. Rep.* 18 2622–2634. 10.1016/j.celrep.2017.02.059 28297667 PMC5479356

[B6] Dai D. Wang H. Zhu L. Jin H. Wang X. (2018). N6-methyladenosine links RNA metabolism to cancer progression. *Cell Death Dis.* 9:124. 10.1038/s41419-017-0129-x PMC5833385 29374143

[B7] Ding J. Zhao J. Huan L. Liu Y. Qiao Y. Wang Z. (2020). Inflammation-induced LINC00665 increases the malignancy through activating PKR/NF-kappaB pathway in hepatocellular carcinoma. *Hepatology* [Epub ahead of print]. 10.1002/hep.31195 32083756

[B8] Feng L. Lin T. Che H. Wang X. (2020). Long noncoding RNA DANCR knockdown inhibits proliferation, migration and invasion of glioma by regulating miR-135a-5p/BMI1. *Cancer Cell Int.* 20:53. 10.1186/s12935-020-1123-4 PMC7029463 32099526

[B9] Friedman J. Hastie T. Tibshirani R. (2010). Regularization paths for generalized linear models via coordinate descent. *J. Stat. Softw.* 33 1–22. 20808728 PMC2929880

[B10] Han Y. Wu Z. Wu T. Huang Y. Cheng Z. Li X. (2016). Tumor-suppressive function of long noncoding RNA MALAT1 in glioma cells by downregulation of MMP2 and inactivation of ERK/MAPK signaling. *Cell Death Dis.* 7:e2123 10.1038/cddis.2015.407 PMC4823926 26938295

[B11] Jiang T. Mao Y. Ma W. Mao Q. You Y. Yang X. (2016). Chinese glioma cooperative, CGCG clinical practice guidelines for the management of adult diffuse gliomas. *Cancer Lett.* 375 263–273. 26966000 10.1016/j.canlet.2016.01.024

[B12] Lan T. Li H. Zhang D. Xu L. Liu H. Hao X. (2019). KIAA1429 contributes to liver cancer progression through N6-methyladenosine-dependent post-transcriptional modification of GATA3. *Mol. Cancer* 18:186. 10.1186/s12943-019-1106-z PMC6921542 31856849

[B13] Liu J. Ren D. Du Z. Wang H. Zhang H. Jin Y. (2018). m(6)A demethylase FTO facilitates tumor progression in lung squamous cell carcinoma by regulating MZF1 expression. *Biochem. Biophys. Res. Commun.* 502 456–464. 10.1016/j.bbrc.2018.05.175 29842885

[B14] Ma L. Kuai W. X. Sun X. Z. Lu X. C. Yuan Y. F. (2018). Long noncoding RNA LINC00265 predicts the prognosis of acute myeloid leukemia patients and functions as a promoter by activating PI3K-AKT pathway. *Eur. Rev. Med. Pharmacol. Sci.* 22 7867–7876. 30536332 10.26355/eurrev_201811_16412

[B15] Ni W. Yao S. Zhou Y. Liu Y. Huang P. Zhou A. (2019). Long noncoding RNA GAS5 inhibits progression of colorectal cancer by interacting with and triggering YAP phosphorylation and degradation and is negatively regulated by the m(6)A reader YTHDF3. *Mol. Cancer* 18:143. 10.1186/s12943-019-1079-y PMC6794841 31619268

[B16] Ou C. Sun Z. He X. Li X. Fan S. Zheng X. (2020). Targeting YAP1/LINC00152/FSCN1 signaling axis prevents the progression of colorectal cancer. *Adv. Sci.* 7:1901380 10.1002/advs.201901380 PMC7001651 32042551

[B17] Ritchie M. E. Phipson B. Wu D. Hu Y. Law C. W. Shi W. (2015). limma powers differential expression analyses for RNA-sequencing and microarray studies. *Nucleic Acids Res.* 43:e47 10.1093/nar/gkv007 PMC4402510 25605792

[B18] Se Y. B. Kim S. H. Kim J. Y. Kim J. E. Dho Y. S. Kim J. W. (2017). Underexpression of HOXA11 is associated with treatment resistance and poor prognosis in glioblastoma. *Cancer Res. Treat.* 49 387–398. 10.4143/crt.2016.106 27456940 PMC5398402

[B19] Seo D. Kim D. Kim W. (2019). Long non-coding RNA linc00152 acting as a promising oncogene in cancer progression. *Genomics Inform.* 17:e36 10.5808/gi.2019.17.4.e36 PMC6944044 31896236

[B20] Shannon P. Markiel A. Ozier O. Baliga N. S. Wang J. T. Ramage D. (2003). Cytoscape: a software environment for integrated models of biomolecular interaction networks. *Genome Res.* 13 2498–2504. 10.1101/gr.1239303 14597658 PMC403769

[B21] Tao C. Huang K. Shi J. Hu Q. Li K. Zhu X. (2020). Genomics and prognosis analysis of epithelial-mesenchymal transition in glioma. *Front. Oncol.* 10:183 10.3389/fonc.2020.00183 PMC7047417 32154177

[B22] The Cancer Genome Atlas (TCGA) Research Network (2008). Comprehensive genomic characterization defines human glioblastoma genes and core pathways. *Nature* 455 1061–1068. 10.1038/nature07385 18772890 PMC2671642

[B23] Wang H. Liu Y. Tang A. (2020). Prognostic values of long noncoding RNA linc00152 in various carcinomas: an updated systematic review and meta-analysis. *Oncologist* 25 e31–e38. 31801898 10.1634/theoncologist.2018-0358 PMC6964117

[B24] Wang Q. Zhang J. Liu Y. Zhang W. Zhou J. Duan R. (2016). A novel cell cycle-associated lncRNA, HOXA11-AS, is transcribed from the 5-prime end of the HOXA transcript and is a biomarker of progression in glioma. *Cancer Lett.* 373 251–259. 10.1016/j.canlet.2016.01.039 26828136

[B25] Weng H. Huang H. Wu H. Qin X. Zhao B. S. Dong L. (2018). METTL14 inhibits hematopoietic stem/progenitor differentiation and promotes leukemogenesis via mRNA m(6)A modification. *Cell Stem Cell* 22 191.e9–205.e9. 29290617 10.1016/j.stem.2017.11.016 PMC5860916

[B26] Yang B. Bai Q. Chen H. Su K. Gao C. (2020). LINC00665 induces gastric cancer progression through activating Wnt signaling pathway. *J. Cell Biochem.* 121 2268–2276. 10.1002/jcb.29449 31736127

[B27] Yang G. Lu X. Yuan L. (2014). LncRNA: a link between RNA and cancer. *Biochim. Biophys. Acta* 1839 1097–1109. 10.1016/j.bbagrm.2014.08.012 25159663

[B28] Yang X. Zhang S. He C. Xue P. Zhang L. He Z. (2020). METTL14 suppresses proliferation and metastasis of colorectal cancer by down-regulating oncogenic long non-coding RNA XIST. *Mol. Cancer* 19:46. 10.1186/s12943-020-1146-4 PMC7047419 32111213

[B29] Zaccara S. Ries R. J. Jaffrey S. R. (2019). Reading, writing and erasing mRNA methylation. *Nat. Rev. Mol. Cell. Biol.* 20 608–624. 10.1038/s41580-019-0168-5 31520073

[B30] Zhang S. Zhao B. S. Zhou A. Lin K. Zheng S. Lu Z. (2017). m(6)A demethylase ALKBH5 maintains tumorigenicity of glioblastoma stem-like cells by sustaining FOXM1 expression and cell proliferation program. *Cancer Cell.* 31 591.e6–606.e6. 28344040 10.1016/j.ccell.2017.02.013 PMC5427719

[B31] Zhao B. S. Roundtree I. A. He C. (2017). Post-transcriptional gene regulation by mRNA modifications. *Nat. Rev. Mol. Cell. Biol.* 18 31–42. 10.1038/nrm.2016.132 27808276 PMC5167638

[B32] Zhong L. Liao D. Zhang M. Zeng C. Li X. Zhang R. (2019). YTHDF2 suppresses cell proliferation and growth via destabilizing the EGFR mRNA in hepatocellular carcinoma. *Cancer Lett.* 442 252–261. 10.1016/j.canlet.2018.11.006 30423408

[B33] Zhou Y. Zhou B. Pache L. Chang M. Khodabakhshi A. H. Tanaseichuk O. (2019). Metascape provides a biologist-oriented resource for the analysis of systems-level datasets. *Nat. Commun.* 10:1523. 10.1038/s41467-019-09234-6 PMC6447622 30944313

[B34] Zhu Y. Gu L. Lin X. Cui K. Liu C. Lu B. (2019). LINC00265 promotes colorectal tumorigenesis via ZMIZ2 and USP7-mediated stabilization of beta-catenin. *Cell Death Diff.* 27 1316–1327. 10.1038/s41418-019-0417-3 PMC7206056 31527801

