# Supplementary figures and images for: N6-Methylandenosine-Related lncRNAs Are Potential Biomarkers for Predicting the Overall Survival of Lower-Grade Glioma Patients
Source: Front Cell Dev Biol. 2020 Jul 23;8:642. doi: 10.3389/fcell.2020.00642 (PMC7390977; doi:10.3389/fcell.2020.00642)

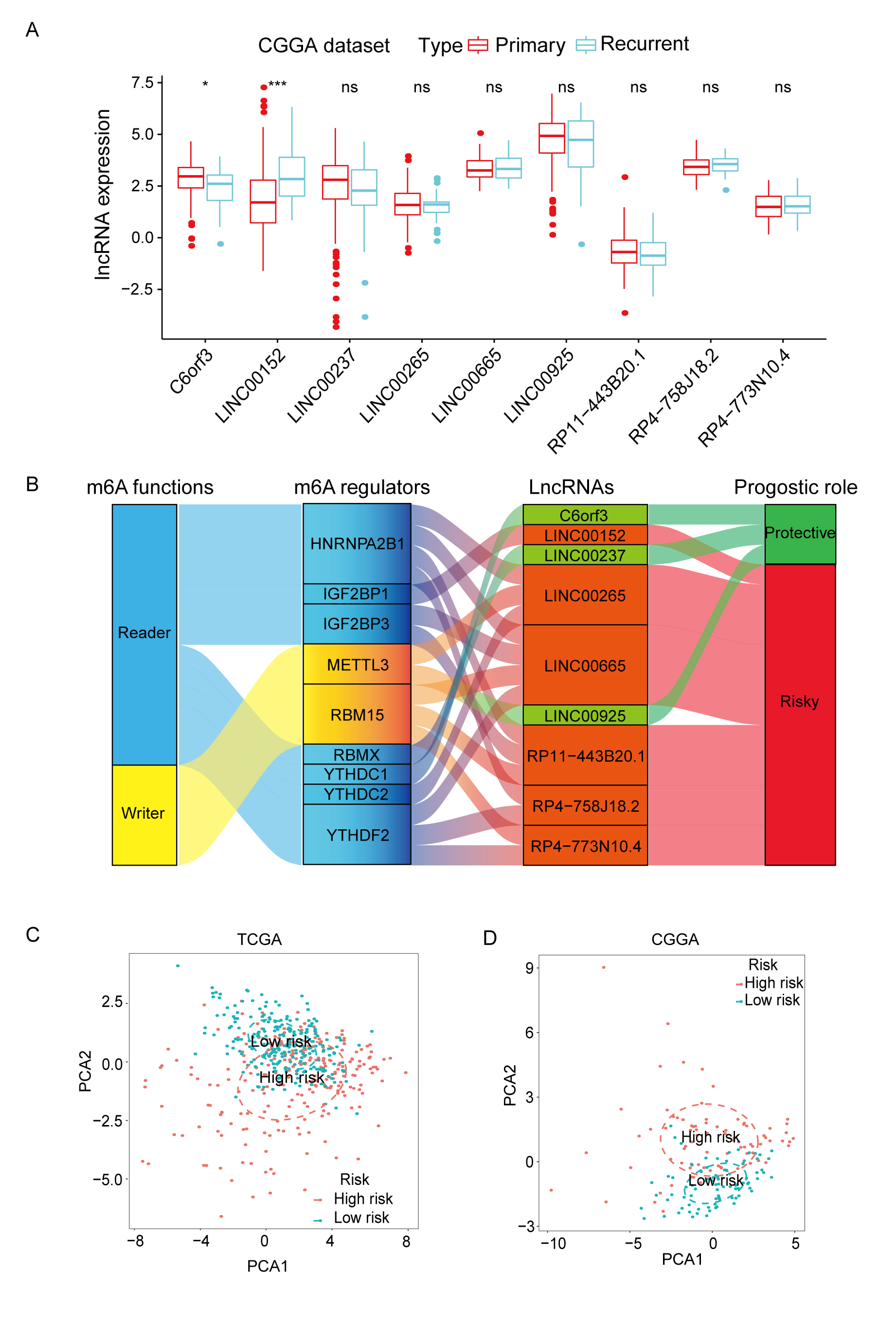

Supplement: Supplementary file 1 [file Image_1.TIF]

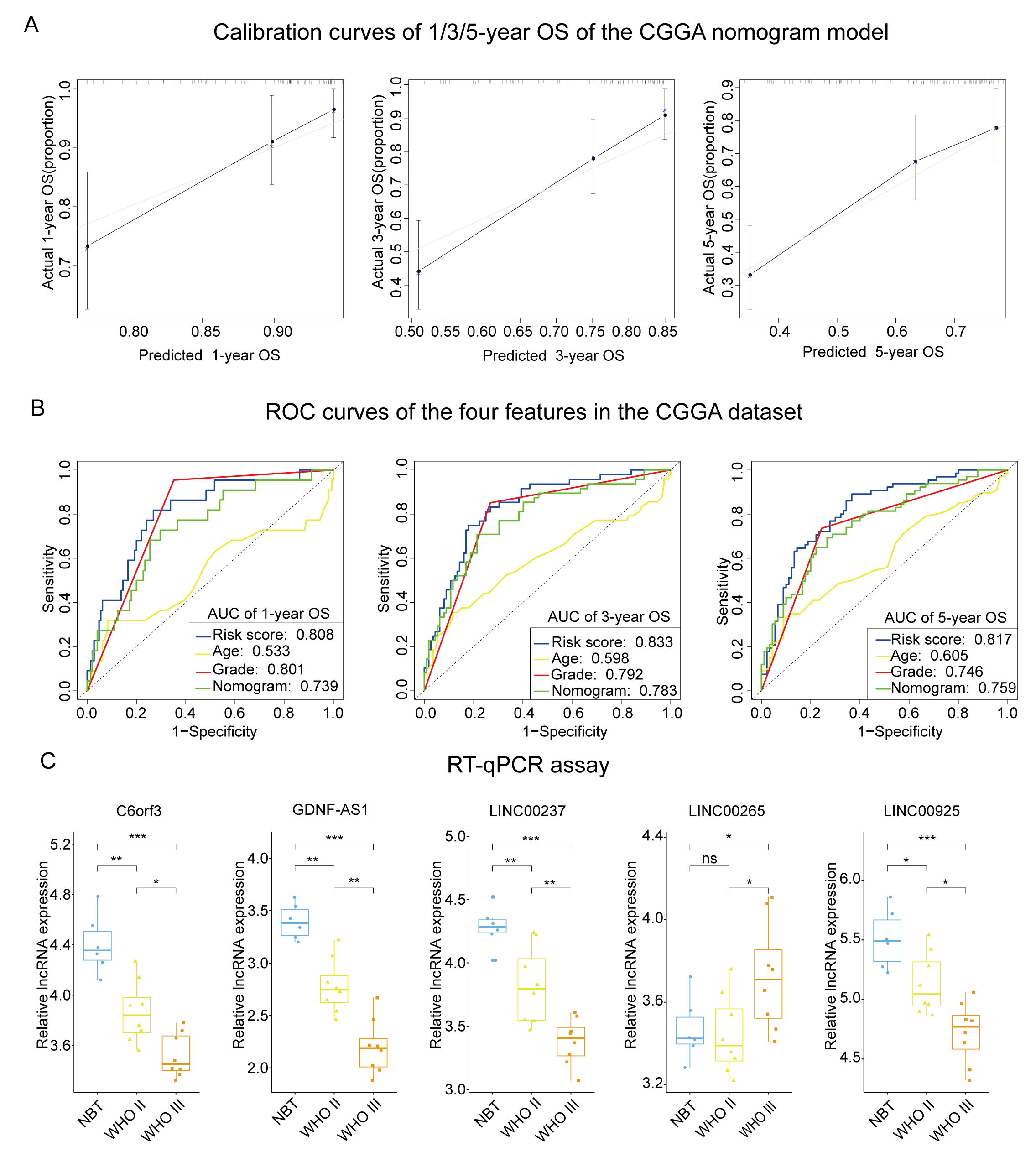

Supplement: Supplementary file 2 [file Image_2.TIF]
