# Supplementary material for: N6-Methylandenosine-Related lncRNAs Are Potential Biomarkers for Predicting the Overall Survival of Lower-Grade Glioma Patients
Source: Front Cell Dev Biol. 2020 Jul 23;8:642. doi: 10.3389/fcell.2020.00642 (PMC7390977; doi:10.3389/fcell.2020.00642)
Supplement: Supplementary file 3 [file Table_1.DOCX]

**Table 1. The twenty-four m6A-related prognostic lncRNAs.**

| **m6A-related lncRNA** | **Biotype** | **TCGA** | | | | **CGGA** | | | |
| --- | --- | --- | --- | --- | --- | --- | --- | --- | --- |
|  |  | **HR** | **HR.95L** | **HR.95H** | **p value** | **HR** | **HR.95L** | **HR.95H** | **p value** |
| **GDNF-AS1** | **lincRNA** | 0.5263 | 0.4094 | 0.6765 | 5.42E-07 | 0.8898 | 0.8093 | 0.9782 | 1.58E-02 |
| **TMCC1-AS1** | **antisense** | 0.6013 | 0.4645 | 0.7784 | 1.13E-04 | 0.8782 | 0.7828 | 0.9852 | 2.68E-02 |
| **LINC00237** | **lincRNA** | 0.8579 | 0.7936 | 0.9274 | 1.15E-04 | 0.9523 | 0.9178 | 0.9881 | 9.49E-03 |
| **C6orf3** | **antisense** | 0.8921 | 0.8522 | 0.9339 | 1.01E-06 | 0.9057 | 0.8539 | 0.9607 | 9.94E-04 |
| **LINC00925** | **lincRNA** | 0.9257 | 0.9039 | 0.9480 | 1.99E-10 | 0.9899 | 0.9801 | 0.9999 | 4.84E-02 |
| **TUG1** | **antisense** | 0.9737 | 0.9515 | 0.9965 | 2.37E-02 | 0.9844 | 0.9710 | 0.9979 | 2.37E-02 |
| **RP4-758J18.2** | **processed transcript** | 1.1490 | 1.0908 | 1.2103 | 1.66E-07 | 1.2195 | 1.1626 | 1.2792 | 3.88E-16 |
| **CTD-2537I9.12** | **antisense** | 1.1511 | 1.0731 | 1.2349 | 8.56E-05 | 1.0814 | 1.0453 | 1.1187 | 6.29E-06 |
| **LINC00339** | **lincRNA** | 1.1819 | 1.1035 | 1.2660 | 1.84E-06 | 1.2501 | 1.1695 | 1.3362 | 5.20E-11 |
| **CTC-444N24.8** | **lincRNA** | 1.1861 | 1.0894 | 1.2913 | 8.34E-05 | 1.4696 | 1.2072 | 1.7891 | 1.25E-04 |
| **RP11-548H3.1** | **antisense** | 1.2135 | 1.0639 | 1.3841 | 3.95E-03 | 1.3994 | 1.2191 | 1.6063 | 1.79E-06 |
| **PTOV1-AS1** | **antisense** | 1.2212 | 1.0478 | 1.4234 | 1.05E-02 | 1.3643 | 1.2400 | 1.5010 | 1.84E-10 |
| **LINC00265** | **lincRNA** | 1.2224 | 1.1064 | 1.3504 | 7.82E-05 | 1.1418 | 1.0475 | 1.2446 | 2.56E-03 |
| **RP4-773N10.4** | **antisense** | 1.2399 | 1.0739 | 1.4315 | 3.36E-03 | 1.6187 | 1.3940 | 1.8795 | 2.66E-10 |
| **AC064875.2** | **processed transcript** | 1.2407 | 1.1629 | 1.3236 | 6.50E-11 | 1.0864 | 1.0413 | 1.1334 | 1.28E-04 |
| **RP4-758J18.13** | **lincRNA** | 1.2553 | 1.0465 | 1.5057 | 1.43E-02 | 1.8533 | 1.5635 | 2.1967 | 1.14E-12 |
| **RP5-1074L1.4** | **antisense** | 1.2631 | 1.0992 | 1.4515 | 9.88E-04 | 1.9899 | 1.5559 | 2.5450 | 4.22E-08 |
| **RP11-1376P16.2** | **antisense** | 1.2936 | 1.0586 | 1.5807 | 1.19E-02 | 1.5986 | 1.1419 | 2.2381 | 6.28E-03 |
| **LINC00665** | **lincRNA** | 1.4320 | 1.2835 | 1.5977 | 1.29E-10 | 1.1387 | 1.0988 | 1.1800 | 9.17E-13 |
| **RP5-997D16.2** | **antisense** | 1.4971 | 1.2160 | 1.8432 | 1.43E-04 | 2.3026 | 1.7693 | 2.9965 | 5.45E-10 |
| **LINC00152** | **lincRNA** | 1.4973 | 1.3795 | 1.6252 | 4.58E-22 | 1.0192 | 1.0109 | 1.0276 | 5.72E-06 |
| **AC010761.8** | **antisense** | 1.5050 | 1.1462 | 1.9763 | 3.27E-03 | 1.9206 | 1.4746 | 2.5015 | 1.30E-06 |
| **RP11-148K1.12** | **antisense** | 1.7822 | 1.3549 | 2.3444 | 3.61E-05 | 1.4744 | 1.2462 | 1.7443 | 6.01E-06 |
| **RP11-443B20.1** | **antisense** | 1.8202 | 1.5599 | 2.1240 | 2.83E-14 | 1.5240 | 1.2881 | 1.8031 | 9.11E-07 |
