# Supplementary material for: N6-Methylandenosine-Related lncRNAs Are Potential Biomarkers for Predicting the Overall Survival of Lower-Grade Glioma Patients
Source: Front Cell Dev Biol. 2020 Jul 23;8:642. doi: 10.3389/fcell.2020.00642 (PMC7390977; doi:10.3389/fcell.2020.00642)
Supplement: Supplementary file 4 [file Table_2.docx]

**Table S2. Clinicopathological features between low- and high-risk subgroups.**

|  | | **TCGA** | | | **CGGA** | | |
| --- | --- | --- | --- | --- | --- | --- | --- |
|  |  | **Low-risk** | **High-risk** | **p value** | **Low-risk** | **High-risk** | **p value** |
| **Total case** |  | 238 | 238 |  | 85 | 85 |  |
| **Age** |  |  |  | 0.099 |  |  | 0.014 |
|  | **<median** | 125 | 106 |  | 48 | 31 |  |
|  | **≥median** | 113 | 132 |  | 37 | 54 |  |
| **Gender** |  |  |  | 1 |  |  | 0.344 |
|  | **Male** | 130 | 130 |  | 49 | 56 |  |
|  | **Female** | 108 | 108 |  | 36 | 29 |  |
| **WHO grade** |  |  |  | <0.001 |  |  | <0.001 |
|  | **II** | 144 | 87 |  | 66 | 31 |  |
|  | **III** | 94 | 151 |  | 19 | 54 |  |
| **IDH status** |  |  |  | <0.001 |  |  | <0.001 |
|  | **Mutant** | 236 | 152 |  | 81 | 44 |  |
|  | **Wild** | 0 | 85 |  | 4 | 40 |  |
|  | **NA** | 2 | 1 |  | 0 | 1 |  |
| **1p/19q status** |  |  |  | <0.001 |  |  | <0.001 |
|  | **Codeletion** | 129 | 27 |  | 46 | 10 |  |
|  | **Non-codeletion** | 109 | 211 |  | 39 | 73 |  |
|  | **NA** | 0 | 0 |  | 0 | 2 |  |
