# Supplementary material for: N6-Methylandenosine-Related lncRNAs Are Potential Biomarkers for Predicting the Overall Survival of Lower-Grade Glioma Patients
Source: Front Cell Dev Biol. 2020 Jul 23;8:642. doi: 10.3389/fcell.2020.00642 (PMC7390977; doi:10.3389/fcell.2020.00642)
Supplement: Supplementary file 5 [file Table_3.docx]

**Table S3. Clinicopathological features of the samples included in this study.**

|  | | **TCGA** | | **CGGA** | |
| --- | --- | --- | --- | --- | --- |
|  |  | **Number** | **Percentage** | **Number** | **Percentage** |
| **Total** |  | 476 | 100.00% | 170 | 100.00% |
| **Age** |  | 14-87(41) |  | 10-74(39) |  |
|  | **<median** | 231 | 48.53% | 79 | 46.47% |
|  | **≥median** | 245 | 51.47% | 91 | 53.53% |
| **Gender** |  |  |  |  |  |
|  | **Male** | 260 | 54.62% | 105 | 61.76% |
|  | **Female** | 216 | 45.38% | 65 | 38.24% |
| **WHO grade** |  |  |  |  |  |
|  | **II** | 231 | 48.53% | 97 | 57.06% |
|  | **III** | 245 | 51.47% | 73 | 42.94% |
| **IDH status** |  |  |  |  |  |
|  | **Mutant** | 388 | 81.51% | 125 | 73.53% |
|  | **Wild** | 85 | 17.86% | 44 | 25.88% |
|  | **NA** | 3 | 0.63% | 1 | 0.59% |
| **1p/19q status** |  |  |  |  |  |
|  | **Codeletion** | 156 | 32.77% | 56 | 32.94% |
|  | **Non-codeletion** | 320 | 67.23% | 112 | 65.88% |
|  | **NA** | 0 | 0.00% | 2 | 1.18% |
